# Germline DNA copy number variation in familial and early-onset breast cancer

**DOI:** 10.1186/bcr3109

**Published:** 2012-02-07

**Authors:** Ana CV Krepischi, Maria Isabel W Achatz, Erika MM Santos, Silvia S Costa, Bianca CG Lisboa, Helena Brentani, Tiago M Santos, Amanda Gonçalves, Amanda F Nóbrega, Peter L Pearson, Angela M Vianna-Morgante, Dirce M Carraro, Ricardo R Brentani, Carla Rosenberg

**Affiliations:** 1 National Institute of Science and Technology in Oncogenomics, AC Camargo Hospital, Rua Taguá 440, 01508-010, São Paulo, Brazil; 2 Department of Genetics and Evolutionary Biology, Institute of Biosciences, University of São Paulo, Rua do Matão 277, 05508-090, São Paulo, Brazil; 3 Department of Psychiatry, Medicine College, University of São Paulo, Rua Dr Ovídio Pires de Campos 785, 01060-970, São Paulo, Brazil; 4 Institute of Mathematics and Statistics, University of São Paulo, Rua do Matão 1010, 05508-090, São Paulo, Brazil

## Abstract

**Introduction:**

Genetic factors predisposing individuals to cancer remain elusive in the majority of patients with a familial or clinical history suggestive of hereditary breast cancer. Germline DNA copy number variation (CNV) has recently been implicated in predisposition to cancers such as neuroblastomas as well as prostate and colorectal cancer. We evaluated the role of germline CNVs in breast cancer susceptibility, in particular those with low population frequencies (rare CNVs), which are more likely to cause disease."

**Methods:**

Using whole-genome comparative genomic hybridization on microarrays, we screened a cohort of women fulfilling criteria for hereditary breast cancer who did not carry *BRCA1/BRCA2 *mutations.

**Results:**

The median numbers of total and rare CNVs per genome were not different between controls and patients. A total of 26 rare germline CNVs were identified in 68 cancer patients, however, a proportion that was significantly different (*P *= 0.0311) from the control group (23 rare CNVs in 100 individuals). Several of the genes affected by CNV in patients and controls had already been implicated in cancer.

**Conclusions:**

This study is the first to explore the contribution of germline CNVs to *BRCA1/2*-negative familial and early-onset breast cancer. The data suggest that rare CNVs may contribute to cancer predisposition in this small cohort of patients, and this trend needs to be confirmed in larger population samples.

## Introduction

It has been estimated that all known cancer susceptibility genes account for only 1% to 15% of familial cancers [1,2]. Approximately 5% to 10% of hereditary breast and ovarian cancers result from dominant mutations in known single genes [3-6], particularly *BRCA1/BRCA2*. Therefore, the basis for a large fraction of genetic predisposition in families with breast and/or ovarian cancer remains to be uncovered.

Recent studies have highlighted DNA copy number variation (CNV) as the most prevalent type of structural variation in the human genome [7-9], and its role in normal development and disease has been demonstrated through its impact on gene expression and protein structure [10-13]. In particular, CNVs involving deletions have been reported as a cause of cancer susceptibility, occurring in up to 30% of highly penetrant cancer-predisposing genes, including *BRCA1, BRCA2, APC, SMAD4 *and *TP53*, as well as mismatch repair genes [14-16] (reviewed in [17,18]).

Germline gains and losses of large DNA segments have recently been reported as factors predisposing individuals to neuroblastoma, prostate and colorectal cancer and *BRCA1*-associated ovarian cancer [19-24]. Nevertheless, whole-genome CNV profiling of patients fulfilling criteria for hereditary breast and ovarian cancer, but without *BRCA1/BRCA2 *mutations, has not been reported. In the present study, we investigated the germline CNV profiles of 68 unrelated familial and early-onset breast cancer patients who were negative for *BRCA1/BRCA2 *mutations, with the aim of detecting new genes contributing to breast and/or ovarian cancer predisposition.

## Materials and methods

### Study approval

The research protocol was approved by the ethics committee of the AC Camargo Cancer Hospital, São Paulo, Brazil (protocol 1175/08), and informed consent was obtained from the subjects.

### Patients

Samples of peripheral blood cells for DNA extraction were collected after informed consent was obtained from 68 women attending the AC Camargo Cancer Hospital prior to any systemic treatment. They were selected for fulfilling at least one of the criteria for hereditary breast and ovarian cancer published in the National Comprehensive Cancer Network Practice Guidelines in Oncology version 1.2010 [25].

Confirmation of the family history of cancer was obtained whenever possible on the basis of pathology reports, medical records and/or death certificates. All women had previously tested negative for *BRCA1/BRCA2 *pathogenic mutations (based on Sanger sequencing of coding sequences). Because most of the affected relatives were already dead, were inaccessible or refused to participate, we were unable to investigate CNV segregation in the majority of the cases.

The criteria used to select patients, type of cancer, and age at cancer diagnosis are given in Additional file 1. Most of the patients (*n *= 48) were familial cases of hereditary breast and/or ovarian cancer in which at least one other family member was affected. The remaining 20 patients were considered hereditary breast and/or ovarian cancer patients for being isolated cases of early-onset cancer (≤ 45 years of age). Most of the tumors were invasive ductal breast carcinomas. Aside from two patients who had only ovarian cancer (patients 9 and 34), all of the other sixty-six patients had breast cancer (bilateral in patient 67, and patients 10 and 16 also had ovarian cancer).

### Control sample

DNA samples were obtained from the peripheral blood cells of control participants after their informed consent was obtained. One hundred DNA samples (seventy-eight women and twenty-two men) were provided by the Genetic Center of the Institute of Biosciences, University of São Paulo, São Paulo, Brazil. They were obtained from noncarrier relatives of patients affected by mental impairment with clear genetic etiology unrelated to cancer predisposition (namely, fragile × syndrome or *de novo *chromosomal rearrangements). No information regarding their cancer history was available. Age-matching of controls and patients was considered unnecessary for this study since CNV frequency in blood is generally considered stable and unrelated to chronological age.

### Comparative genomic hybridization based on microarray (array-CGH)

We performed comparative genomic hybridization based on microarray (array-CGH) using a 180 K whole-genome platform (design 22060; Agilent Technologies, Santa Clara, CA, USA), which has an average probe spacing of 18 kb. Briefly, samples were labeled with Cy3- and Cy5-deoxycytidine triphosphates by random priming. Purification, hybridization and washing were carried out as previously reported [26,27]. Scanned images of the arrays were processed using Feature Extraction software (Agilent Technologies).

We applied the Genomic Workbench software (Agilent Technologies) for calling DNA CNV using the aberration detection method 2 statistical algorithm with a sensitivity threshold of 6.7. Poor quality hybridization (QC > 0.3) was disregarded. Duplication or deletion of genomic segments was considered when the log_2 _ratio of the Cy3/Cy5 intensities of a given region encompassing at least three probes was > 0.3 or < -0.3, respectively. All hybridizations were gender-matched and processed in reverse-labeling duplicates as described previously [28]. CNVs that were not detected in both experiments were disregarded.

### Copy number validation by real-time PCR

Selected CNVs detected in the patient group were validated by real-time quantitative PCR (qPCR) [29] using the SYBR Green system (Roche Applied Science, Indianapolis, IN, USA) on a 7500 Fast Real-Time PCR System apparatus (Applied Biosystems, Foster City, CA, USA). As controls or for copy number calibration, we used three DNA samples obtained from healthy donors and the qPCR values for the *GAPD *and *HPRT *genes for normalization. All samples were run in duplicate, and the data were analyzed with Microsoft Excel software (Microsoft Corp, Redmond, WA, USA) using the comparative ΔΔ^C^_t _cycle threshold method (Applied Biosystems), which assumes that the calibrator DNA has two copies of the control genes.

### Data analysis

Detected CNVs were compared to CNV data from oligoarray studies documented in the Database of Genomic Variants (DGV) [30]. We classified the CNVs into "rare" and "common," with rare being those that encompassed coding sequences which had never been documented as variable in the general population (DGV). CNVs were evaluated regarding proportion of total and rare CNVs, frequency of deletions and duplications, length, and gene content using the Mann-Whitney *U *test and Fisher's exact test.

Gene annotation was performed using the University of California Santa Cruz Genome Browser (UCSC) [31], BioMart in the Ensembl Genome Browser [32] and Catalog of Somatic Mutations. We investigated the biological features of genes contained within the rare CNVs using GOTree Machine (GOTM) software [33] to measure the enrichment in the Gene Ontology (GO) and Kyoto Encyclopedia of Genes and Genomes (KEGG) categories. GOTM reports only those enrichments that are statistically significant as determined by the hypergeometric test [34].

## Results

Full CNV data on the controls and patients can be found in Additional file 2. A whole extra copy of the × chromosome was identified in one patient. This chromosomal numerical alteration was not considered a CNV. The array-CGH results are summarized in Table 1. We found a total of 1,238 CNVs in 168 individuals. CNVs observed in both patient and control samples corresponded to 81.3% of the total, all of which overlapped common CNVs (DGV). CNVs detected exclusively in one of the groups corresponded to 110 events in patients and 121 in controls. The distribution of CNVs did not differ between patients and controls (*P *= 0.1724, Mann-Whitney *U *test).

**Table 1:** Summary of DNA copy number variation data from breast and/or ovarian cancer patients and controls^a^

Copy number variation	Controls (*n *= 100)	Patients (*n *= 68)
Total CNVs	702	536
Rare variants	23	26
Deletions	9	12
Duplications	14	14
Median CNVs per individual (IQR)^b^	7.0 (4 to 9)	7.5 (5 to 10)

^a^CNV = copy number variation. ^b^IQR = interquartile range.

Only 49 of the 1,238 CNVs could be classified as rare, and none of them were recurrent. Those CNVs classified as rare based on DGV data corresponded to 4% (49 of 1,238 CNVs) of all CNVs detected in our study. The log_2 _ratios of the rare CNVs (all outside the log_2 _-0.65 to log_2 _0.45 range) were not suggestive of mosaicism, indicating that these CNVs are likely constitutive. In the control group, 3.28% (23 of 702) of CNVs detected in 23 of 100 individuals (23%) were classified as rare, whereas 26 of 536 rare CNVs (4.85%) were found in 25 of 68 patients (37%). The median numbers of total and rare CNVs per genome did not differ between controls and patients. However, the proportion of rare CNVs in patients was higher than in the controls (*P *= 0.0311, Fisher's exact test). The relative frequencies of duplications and deletions among rare CNVs were similar between controls (14 duplications and 9 deletions) and patients (14 duplications and 12 deletions).

We also inspected the rare CNVs for length and gene content (Table 2). The length of rare CNVs in controls and patients did not differ significantly. The lengths of rare deletions or duplications did not differ between patients and controls or within the patient group alone. However, only in the control group were the deletions found to be significantly smaller than the duplications (*P *= 0.0089, Mann-Whitney *U *test).

**Table 2:** Size and gene content of rare copy number variations^a^

Rare CNVs	Control group (*n *= 23 CNVs)	Patient group (*n *= 26 CNVs)
Length range (kb)	31 to 684	32 to 1,592
Median size (IQR)^b ^(kb)	225.5 (125.8 to 275.3)	136.7 (70.3 to 278.0)
Deletions (kb)	127.2 (43.7 to 225.5)	145.0 (124.6 to 229.5)
Duplications (kb)	249.5 (202.9.4 to 350.4)	119.2 (50.0 to 278.0)
Gene number/individual	0.4 (44 of 100)	0.8 (57 of 68)

^a^CNV = copy number variation. ^b^IQR = interquartile range.

The 26 rare CNVs and the affected genes detected in patients are described in Table 3. The published literature regarding those genes already reported to be altered in cancer is listed in Additional file 3. The rare CNVs identified in the control group are listed in Additional file 4. To evaluate whether the rare CNVs detected among cancer patients represent common CNVs in the Brazilian population, we compiled CNV data obtained from independent samples studied in our laboratory using 180 K array-CGH. These individuals (120 females and 38 males) were selected on the basis of criteria other than cancer history, including 52 patients with dementia, 56 cases of nonsyndromic hearing loss and 51 cases of Müllerian anomalies (C Rosenberg; ACV Krepischi; unpublished data). None of the rare CNVs documented in this study were detected in these independent cohorts of patients.

**Table 3:** Genomic positions (build 36-Hg18), size, type and affected genes of the 26 rare copy number variations identified in patients^a^

Chromosome	Cytoband	Start site	Size (bp)	CNV type	Gene Names	Patient
chr1	p31.1	76,801,602	550,723	del	*ST6GALNAC3, ST6GALNAC5, PIGK*	3
chr1	q44	243,610,556	153,230	del	*KIF26B*	16
chr1	p32.1	59,559,568	41,339	del	*FGGY*	15
chr2	p25.1	9,165,985	192,606	del	*ASAP2*	21
chr2	q22.2	143,463,273	300,408	dup	*KYNU, ARHGAP15*	7
chr2	q32.2	190,015,149	45,366	dup	*WDR75*	28
chr3	p24.3	18,759,412	635,060	dup	*KCNH8, MIR4791*	11
chr3	q28	193,405,249	135,458	dup	*FGF12*	1
chr4	q31.3	152,508,270	136,605	del	*FAM160A1*	29
chr6	p12.1	55,468,168	33,031	dup	*HMGCLL1*	30
chr9	p21.3	20,661,515	136,814	del	*KIAA1797, MIR491*	13
chr9	p24.1	5,130,196	168,153	del	*INSL6, INSL4, RLN2*	20
chr9	q31.3	111,832,790	340,164	del	*PALM2-AKAP2, AKAP2, C9orf152, TXN, TXNDC8, SVEP1*	22
chr10	p13	16,970,291	103,018	dup	*CUBN*	19
chr11	q12.3	62,290,497	38,091	dup	*POLR2G, TAF6L, TMEM179B, TMEM223, NXF1*	25
chr16	q23.3	81,315,382	136,618	del	*CDH13*	5
chr16	q11.2	45,058,042	198,742	dup	*ANKRD26P1, SHCBP1, VPS35*	6
chr17	q25.1	71,478,161	33,362	dup	*ACOX1, TEN1, CDK3*	5
chr18	q12.1	27,990,329	64,132	dup	*MEP1B*	18
chr21	q21.3	29,391,572	496,425	del	*C21orf7, LINC00189, BACH1, GRIK1*	8
chr21	q22.3	46,538,913	210,594	dup	*YBEY, C21orf58, PCNT, DIP2A*	12
chrX	q22.3	109,193,988	31,659	del	*TMEM164, MIR3978*	23
chrX	q25	126,971,799	88,694	del	*ACTRT1*	17
chrX	q13.1	68,398,248	639,366	dup	*FAM155B, EDA*	27
chrX	p22.31	6,499,677	1,592,274	dup	*HDHD1, STS, VCX, PNPLA4*	15
chrX	q13.3	75,294,586	88,796	dup	*CXorf26*	14

^a^CNV = copy number variation; del = deletion; dup = duplication.

**Figure 1:**
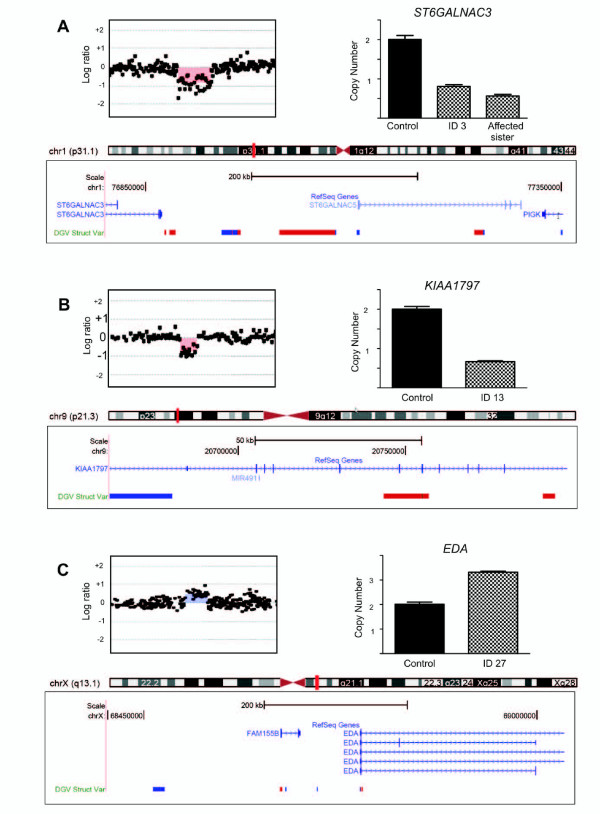
**Rare germline copy number variations detected in three unrelated breast cancer patients**. **(A) **to **(C) **Top panels exhibit the array comparative genomic hybridization profiles of the chromosome regions (images based on the Genomic Workbench software) containing the selected copy number variation (CNV) (left) and the copy number of the corresponding sequences assessed by quantitative PCR in patients 3, 13 and 27 and control individuals (right). Bottom panels show the CNV positions in the chromosomes (small vertical red bars in the ideograms) and the corresponding genomic segments. The encompassed genes according to the Reference Sequence (RefSeq) collection are indicated by blue lines in the RefSeq genes track, and the CNVs *loci *reported in the general population are indicated by the blue (gain) and red (loss) bars in the Database of Genomic Variants (DGV) Struct Var track (data retrieved from the DGV) (images derived from the UCSC Genome Browser, freeze October 2011). Part (A) shows microdeletion at 1p31.1 of a 540 kb genomic segment containing the sequences of three genes. This alteration cosegregated with early-onset breast cancer in patient 3 and her sister. Part (B) shows microdeletion at 9p21.3 of a 137 kb genomic segment encompassing the gene *KIA1797 *and the microRNA *MIR491 *(patient 13). Part (C) shows microduplication at Xq13.1 of a 640 kb genomic segment affecting the *FAM155B *and *EDA *genes (patient 27).

In one family, the patient with early-onset breast cancer (patient 3), as well as her affected sister, who presented with breast cancer at 28 years of age, were found to carry a 540 kb 1p31.1 microdeletion (Figure 1A). The heterozygous deletion was confirmed in the two affected sisters by qPCR. In the second family, a 90 kb microdeletion at Xq25 was detected in two affected sisters, one of whom presented with bilateral breast cancer (patient 17).

Figure 1 depicts two additional rare CNVs identified by array-CGH and validated by qPCR in unrelated patients. Figure 1B shows a 137 kb deletion at 9p21.3 detected in patient 13. Figure 1C illustrates a 640-kb duplication mapped at Xq13.1 (patient 27).

We characterized the function of genes located in rare CNVs in both patients and controls by GO term and KEGG pathway analysis using the Gene Ontology Tree Machine. We did not detect any significant difference in gene content in either patients or controls.

## Discussion

We used array-CGH to investigate the role of rare germline CNVs in probands of individuals with a familial history of breast and ovarian cancer. Because evaluation of CNV profiles depends on ethnic background, array platform and method of analysis [35-38], all experiments were performed using the same platform, the same analytical parameters and a Brazilian control group. We disregarded the possibility that the CNVs were somatically acquired because none of the results were suggestive of mosaicism. Furthermore, the limited data available indicate that CNV profiles are rather stable in adult tissues (reviewed in [39]).

Common CNVs often contain cancer-related genes and likely play a role in carcinogenesis [40]. However, only a minority of CNVs, those with low population frequencies (rare CNVs), would be likely to contain genes that are highly penetrant genetic factors for disease susceptibility, including cancer [7,41]. In our study, the median numbers of total and rare CNVs per genome were quite similar in patients and controls, reflecting a lack of genomic instability in this cohort of patients. These results are in agreement with data derived from a study of *BRCA1*-associated ovarian cancer patients [42]. Nevertheless, the patients did present a higher proportion of rare CNVs compared to controls. None of these rare CNVs were present in an independent cohort of more than 150 individuals, providing support for their nonpolymorphic nature in the Brazilian population. Assuming that some of these rare CNVs are cancer-related, the patients would carry an increased cancer risk proportionate to the number of rare genomic imbalances. The reason why we found a greater proportion of rare CNVs in patients than in controls is not clear. We could speculate that deleterious CNVs tend to be eliminated and, for some reason, conceivably less efficient apoptosis or DNA repair mechanisms, this selection would be less stringent in these patients. Whatever the reason may be, the connection between this finding and the patients' phenotypes deserves investigation.

Part of the rare genomic imbalances harbors genes that could potentially affect cancer susceptibility (see Table 3). For example, a 540 kb microdeletion at 1p31.1 was detected in two affected sisters (Figure 1A). Among the genes mapped to this deleted segment, the most relevant to cancer progression is probably *ST6GALNAC5*, a sialyltransferase recently reported to mediate breast cancer metastasis to the brain [43]. Another interesting alteration detected in a patient, an approximately 137 kb deletion at 9p21.3, encompassed the *KIA1797 *and the *MIR491 *genes (Figure 1B). A germline CNV affecting this genomic segment was recently reported in a colorectal cancer cohort [23]. The finding of a similar 9p21.3 deletion in independent cohorts of cancer patients strengthens their pathogenic role in cancer predisposition.

Our data support the hypothesis that germline DNA CNV is a genetic factor contributing to breast cancer predisposition, which is in accord with the findings of other studies indicating CNVs as risk factors in cancer, including neuroblastoma [19], colorectal cancer [22,23], hepatocellular carcinoma [44], aggressive prostate cancer [20], nasopharyngeal carcinoma[45]and *BRCA1*-associated ovarian cancer[38]. Our findings of a possible association of a cancer predisposition phenotype with rare CNVs affecting different genes are in line with the genetic heterogeneity reported in breast cancer. This picture is different from most of the aforementioned studies, which detected recurrent common CNVs associated with cancer risk, except for prostate, colorectal and ovarian cancer CNV studies, which exhibited high CNV heterogeneity.

## Conclusions

Our analysis of rare CNVs in a small cohort of *BRCA1/BRCA2 *mutation-negative breast and/or ovarian cancer families suggests an intriguing excess in the proportion of rare CNVs compared to controls. The future challenge will be to expand sample sizes and to follow cosegregation of given CNVs with cancer phenotype within families to identify which of the genes involved in the rare CNVs might contribute to familial breast cancer predisposition.

## Abbreviations

bp: base pair; CNV: copy number variation; array-CGH: comparative genomic hybridization on microarray; kb: kilobase; DGV: Database of Genomic Variants; UCSC: University of California Santa Cruz.

## Competing interests

The authors declare that they have no competing interests.

## Authors' contributions

ACVK participated in the design of the study, performed part of the molecular genetics analysis and drafted the manuscript. SSC carried out part of the array-CGH experiments. BCGL and DMC carried out the screening for *BRCA1/2 *mutations. AG carried out the real-time PCR assays for CNV validation. MIWA was the physician responsible for the clinical trial and the selection and classification of the families and also revised the manuscript. AFN and EMMS participated in the clinical trial and the classification of the families. HB and TMS performed the statistical analysis. RRB and AMVM participated in the design of the study and revised the manuscript. PLP and CR revised the manuscript critically gave their final approval of the version to be published.

## Supplementary Material

Additional file 1 **Supplementary material 1**. Description: Clinical parameters of the patients studied, including age at cancer diagnosis, tumor subtype and grade, tumor node metastasis (TNM) stage and immunohistochemistry data. Click here for file

Additional file 2 **Supplementary material 2**. Description: Full copy number variation (CNV) data of controls and patients (rare CNVs in bold). Chromosome coordinates are given according to Hg18. Click here for file

Additional file 3 **Supplementary material 3**. Description: Literature review of the genes encompassed by rare copy number variations (CNVs) identified in the patients and reported to be altered in cancer. Click here for file

Additional file 4 **Supplementary material 4**. Description: Genomic positions (build 36-Hg18), mapping, type and affected genes of the 23 rare copy number variations identified in controls. Click here for file

## References

[B1] Fearon ER Human cancer syndromes: clues to the origin and nature of cancer Science 1997 278 1043 1050 10.1126/science.278.5340.1043 9353177

[B2] Lichtenstein P Holm NV Verkasalo PK Iliadou A Kaprio J Koskenvuo M Pukkala E Skytthe A Hemminki K Environmental and heritable factors in the causation of cancer: analyses of cohorts of twins from Sweden, Denmark, and Finland N Engl J Med 2000 343 78 85 10.1056/NEJM200007133430201 10891514

[B3] Chompret A [Clinical and molecular diagnosis of inherited breast-ovarian cancer] [in French] J Gynecol Obstet Biol Reprod (Paris) 2003 32 101 119 12717301

[B4] Easton D Familial risks of cancer Eur J Cancer 1999 35 1043 1045 10533446 10.1016/s0959-8049(99)00118-5

[B5] Foulkes WD Inherited susceptibility to common cancers N Engl J Med 2008 359 2143 2153 10.1056/NEJMra0802968 19005198

[B6] Hirshfield KM Rebbeck TR Levine AJ Germline mutations and polymorphisms in the origins of cancers in women J Oncol 2010 2010 297671 20111735 10.1155/2010/297671 PMC2810468

[B7] Conrad DF Pinto D Redon R Feuk L Gokcumen O Zhang Y Aerts J Andrews TD Barnes C Campbell P Fitzgerald T Hu M Ihm CH Kristiansson K Macarthur DG MacDonald JR Onyiah I Pang AW Robson S Stirrups K Valsesia A Walter K Wei J Wellcome Trust Case Control Consortium Tyler-Smith C Carter NP Lee C Scherer SW Hurles ME Origins and functional impact of copy number variation in the human genome Nature 2010 464 704 712 10.1038/nature08516 19812545 PMC3330748

[B8] Iafrate AJ Feuk L Rivera MN Listewnik ML Donahoe PK Qi Y Scherer SW Lee C Detection of large-scale variation in the human genome Nat Genet 2004 36 949 951 10.1038/ng1416 15286789

[B9] Sebat J Lakshmi B Troge J Alexander J Young J Lundin P Månér S Massa H Walker M Chi M Navin N Lucito R Healy J Hicks J Ye K Reiner A Gilliam TC Trask B Patterson N Zetterberg A Wigler M Large-scale copy number polymorphism in the human genome Science 2004 305 525 528 10.1126/science.1098918 15273396

[B10] Feuk L Carson AR Scherer SW Structural variation in the human genome Nat Rev Genet 2006 7 85 97 16418744 10.1038/nrg1767

[B11] Ng PC Levy S Huang J Stockwell TB Walenz BP Li K Axelrod N Busam DA Strausberg RL Venter JC Genetic variation in an individual human exome PLoS Genet 2008 4 e1000160 10.1371/journal.pgen.1000160 18704161 PMC2493042

[B12] Stankiewicz P Lupski JR Structural variation in the human genome and its role in disease Annu Rev Med 2010 61 437 455 10.1146/annurev-med-100708-204735 20059347

[B13] Stranger BE Forrest MS Dunning M Ingle CE Beazley C Thorne N Redon R Bird CP de Grassi A Lee C Tyler-Smith C Carter N Scherer SW Tavaré S Deloukas P Hurles ME Dermitzakis ET Relative impact of nucleotide and copy number variation on gene expression phenotypes Science 2007 315 848 853 10.1126/science.1136678 17289997 PMC2665772

[B14] Bunyan DJ Eccles DM Sillibourne J Wilkins E Thomas NS Shea-Simonds J Duncan PJ Curtis CE Robinson DO Harvey JF Cross NC Dosage analysis of cancer predisposition genes by multiplex ligation-dependent probe amplification Br J Cancer 2004 91 1155 1159 10.1038/sj.bjc.6602121 15475941 PMC2747696

[B15] Casilli F Tournier I Sinilnikova OM Coulet F Soubrier F Houdayer C Hardouin A Berthet P Sobol H Bourdon V Muller D Fricker JP Capoulade-Metay C Chompret A Nogues C Mazoyer S Chappuis P Maillet P Philippe C Lortholary A Gesta P Bézieau S Toulas C Gladieff L Maugard CM Provencher DM Dugast C Delvincourt C Nguyen TD Faivre L The contribution of germline rearrangements to the spectrum of *BRCA2 *mutations J Med Genet 2006 43 e49 10.1136/jmg.2005.040212 16950820 PMC2564582

[B16] Petrij-Bosch A Peelen T van Vliet M van Eijk R Olmer R Drüsedau M Hogervorst FB Hageman S Arts PJ Ligtenberg MJ Meijers-Heijboer H Klijn JG Vasen HF Cornelisse CJ van 't Veer LJ Bakker E van Ommen GJ Devilee P *BRCA1 *genomic deletions are major founder mutations in Dutch breast cancer patients Nat Genet 1997 17 341 345 10.1038/ng1197-341 9354803

[B17] Kuiper RP Ligtenberg MJ Hoogerbrugge N Geurts van Kessel A Germline copy number variation and cancer risk Curr Opin Genet Dev 2010 20 282 289 10.1016/j.gde.2010.03.005 20381334

[B18] Shlien A Baskin B Achatz MI Stavropoulos DJ Nichols KE Hudgins L Morel CF Adam MP Zhukova N Rotin L Novokmet A Druker H Shago M Ray PN Hainaut P Malkin D A common molecular mechanism underlies two phenotypically distinct 17p13.1 microdeletion syndromes Am J Hum Genet 2010 87 631 642 10.1016/j.ajhg.2010.10.007 21056402 PMC2978979

[B19] Diskin SJ Hou C Glessner JT Attiyeh EF Laudenslager M Bosse K Cole K Mossé YP Wood A Lynch JE Pecor K Diamond M Winter C Wang K Kim C Geiger EA McGrady PW Blakemore AI London WB Shaikh TH Bradfield J Grant SF Li H Devoto M Rappaport ER Hakonarson H Maris JM Copy number variation at 1q21.1 associated with neuroblastoma Nature 2009 459 987 991 10.1038/nature08035 19536264 PMC2755253

[B20] Liu W Sun J Li G Zhu Y Zhang S Kim ST Sun J Wiklund F Wiley K Isaacs SD Stattin P Xu J Duggan D Carpten JD Isaacs WB Grönberg H Zheng SL Chang BL Association of a germ-line copy number variation at 2p24.3 and risk for aggressive prostate cancer Cancer Res 2009 69 2176 2179 10.1158/0008-5472.CAN-08-3151 19258504 PMC2743179

[B21] Lucito R Suresh S Walter K Pandey A Lakshmi B Krasnitz A Sebat J Wigler M Klein AP Brune K Palmisano E Maitra A Goggins M Hruban RH Copy-number variants in patients with a strong family history of pancreatic cancer Cancer Biol Ther 2007 6 1592 1599 10.4161/cbt.6.10.4725 17912030

[B22] Thean LF Loi C Ho KS Koh PK Eu KW Cheah PY Genome-wide scan identifies a copy number variable region at 3q26 that regulates *PPM1L *in APC mutation-negative familial colorectal cancer patients Genes Chromosomes Cancer 2010 49 99 106 19847890 10.1002/gcc.20724

[B23] Venkatachalam R Verwiel ET Kamping EJ Hoenselaar E Görgens H Schackert HK van Krieken JH Ligtenberg MJ Hoogerbrugge N van Kessel AG Kuiper RP Identification of candidate predisposing copy number variants in familial and early-onset colorectal cancer patients Int J Cancer 2011 129 1635 1642 10.1002/ijc.25821 21128281

[B24] Yoshihara K Tajima A Adachi S Quan J Sekine M Kase H Yahata T Inoue I Tanaka K Germline copy number variations in *BRCA1*-associated ovarian cancer patients Genes Chromosomes Cancer 2011 50 167 177 10.1002/gcc.20841 21213370

[B25] National Comprehensive Cancer Network http://www.nccn.org/

[B26] Krepischi-Santos AC Rajan D Temple IK Shrubb V Crolla JA Huang S Beal S Otto PA Carter NP Vianna-Morgante AM Rosenberg C Constitutional haploinsufficiency of tumor suppressor genes in mentally retarded patients with microdeletions in 17p13.1 Cytogenet Genome Res 2009 125 1 7 10.1159/000218743 19617690 PMC2813804

[B27] Krepischi AC Knijnenburg J Bertola DR Kim CA Pearson PL Bijlsma E Szuhai K Kok F Vianna-Morgante AM Rosenberg C Two distinct regions in 2q24.2-q24.3 associated with idiopathic epilepsy Epilepsia 2010 51 2457 2460 10.1111/j.1528-1167.2010.02742.x 21204806

[B28] Allemeersch J Van Vooren S Hannes F De Moor B Vermeesch JR Moreau Y An experimental loop design for the detection of constitutional chromosomal aberrations by array CGH BMC Bioinformatics 2009 10 380 10.1186/1471-2105-10-380 19925645 PMC2791104

[B29] Livak KJ Schmittgen TD Analysis of relative gene expression data using real-time quantitative PCR and the 2^-ΔΔ*C*^_T _method Methods 2001 25 402 408 10.1006/meth.2001.1262 11846609

[B30] Database of Genomic Variants http://projects.tcag.ca/variation/

[B31] UCSC Genome Browser http://genome.ucsc.edu/

[B32] BioMart http://www.ensembl.org/biomart/martview/a4c96f57a539d313f74223bb4719aedf

[B33] Gene Ontology Tree Machine software, Gene Set Analysis Toolkit version 2 http://bioinfo.vanderbilt.edu/webgestalt/

[B34] Zhang B Schmoyer D Kirov S Snoddy J GOTree Machine (GOTM): a web-based platform for interpreting sets of interesting genes using Gene Ontology hierarchies BMC Bioinformatics 2004 5 16 10.1186/1471-2105-5-16 14975175 PMC373441

[B35] Jakobsson M Scholz SW Scheet P Gibbs JR VanLiere JM Fung HC Szpiech ZA Degnan JH Wang K Guerreiro R Bras JM Schymick JC Hernandez DG Traynor BJ Simon-Sanchez J Matarin M Britton A van de Leemput J Rafferty I Bucan M Cann HM Hardy JA Rosenberg NA Singleton AB Genotype, haplotype and copy-number variation in worldwide human populations Nature 2008 451 998 1003 10.1038/nature06742 18288195

[B36] Perry GH Ben-Dor A Tsalenko A Sampas N Rodriguez-Revenga L Tran CW Scheffer A Steinfeld I Tsang P Yamada NA Park HS Kim JI Seo JS Yakhini Z Laderman S Bruhn L Lee C The fine-scale and complex architecture of human copy-number variation Am J Hum Genet 2008 82 685 695 10.1016/j.ajhg.2007.12.010 18304495 PMC2661628

[B37] Pinto D Darvishi K Shi X Rajan D Rigler D Fitzgerald T Lionel AC Thiruvahindrapuram B MacDonald JR Mills R Prasad A Noonan K Gribble S Prigmore E Donahoe PK Smith RS Park JH Hurles ME Carter NP Lee C Scherer SW Feuk L Comprehensive assessment of array-based platforms and calling algorithms for detection of copy number variants Nat Biotechnol 2011 29 512 520 10.1038/nbt.1852 21552272 PMC3270583

[B38] White SJ Vissers LE Geurts van Kessel A de Menezes RX Kalay E Lehesjoki AE Giordano PC van de Vosse E Breuning MH Brunner HG den Dunnen JT Veltman JA Variation of CNV distribution in five different ethnic populations Cytogenet Genome Res 2007 118 19 30 10.1159/000106437 17901696

[B39] Mkrtchyan H Gross M Hinreiner S Polytiko A Manvelyan M Mrasek K Kosyakova N Ewers E Nelle H Liehr T Bhatt S Thoma K Gebhart E Wilhelm S Fahsold R Volleth M Weise A The human genome puzzle: the role of copy number variation in somatic mosaicism Curr Genomics 2010 11 426 431 10.2174/138920210793176047 21358987 PMC3018723

[B40] Shlien A Malkin D Copy number variations and cancer susceptibility Curr Opin Oncol 2010 22 55 63 10.1097/CCO.0b013e328333dca4 19952747

[B41] Korbel JO Urban AE Grubert F Du J Royce TE Starr P Zhong G Emanuel BS Weissman SM Snyder M Gerstein MB Systematic prediction and validation of breakpoints associated with copy-number variants in the human genome Proc Natl Acad Sci USA 2007 104 10110 10115 10.1073/pnas.0703834104 17551006 PMC1891248

[B42] Yoshihara K Tajima A Adachi S Quan J Sekine M Kase H Yahata T Inoue I Tanaka K Germline copy number variations in *BRCA1*-associated ovarian cancer patients Genes Chromosomes Cancer 2011 50 167 177 10.1002/gcc.20841 21213370

[B43] Bos PD Zhang XH Nadal C Shu W Gomis RR Nguyen DX Minn AJ van de Vijver MJ Gerald WL Foekens JA Massagué J Genes that mediate breast cancer metastasis to the brain Nature 2009 459 1005 1009 10.1038/nature08021 19421193 PMC2698953

[B44] Clifford RJ Zhang J Meerzaman DM Lyu MS Hu Y Cultraro CM Finney RP Kelley JM Efroni S Greenblum SI Nguyen CV Rowe WL Sharma S Wu G Yan C Zhang H Chung YH Kim JA Park NH Song IH Buetow KH Genetic variations at loci involved in the immune response are risk factors for hepatocellular carcinoma Hepatology 2010 52 2034 2043 10.1002/hep.23943 21105107 PMC8259333

[B45] Tse KP Su WH Yang ML Cheng HY Tsang NM Chang KP Hao SP Yao SY Chang YS A gender-specific association of CNV at 6p21.3 with NPC susceptibility Hum Mol Genet 2011 20 2889 2896 10.1093/hmg/ddr191 21536588 PMC3146013

