# Supplementary material for: Germline DNA copy number variation in familial and early-onset breast cancer
Source: Breast Cancer Res. 2012 Feb 7;14(1):R24. doi: 10.1186/bcr3109 (PMC3496142; doi:10.1186/bcr3109)
Supplement: Additional file 3 — Supplementary material 3. Description: Literature review of the genes encompassed by rare copy number variations (CNVs) identified in the patients and reported to be altered in cancer. [file bcr3109-S3.DOCX]

**Supplementary Material 3** - Literature review of the genes encompassed by rare CNVs identified in the patients and reported to be altered in cancer.

| **Chr** | **Cytoband** | **CNV type** | **Gene Names** | **References** |
| --- | --- | --- | --- | --- |
| chr1 | p31.1 | Del | ST6GALNAC3, ***ST6GALNAC5***, PIGK | [1,2] |
| chr1 | q44 | Del | *KIF26B* | [3] |
| chr2 | p25.1 | Del | *ASAP2* | [4] |
| chr3 | p24.3 | Dup | *KCNH8* | [5] |
| chr3 | q28 | Dup | *FGF12* | [6] |
| chr9 | p21.3 | Del | *KIAA1797, MIR491* | [7,8] |
| chr9 | p24.1 | Del | INSL6**, *INSL4, RLN2*** | [9-14] |
| chr9 | q31.3 | Del | PALM2-AKAP2, AKAP2, C9orf152, ***TXN*,** TXNDC8 | [15,16] |
| chr16 | q23.3 | Del | ***CDH13*** | [17-20] |
| chr16 | q11.2 | Dup | FLJ43980*, SHCBP1*, VPS35 | [21] |
| chr17 | q25.1 | Dup | ACOX1, LOC100134934, *CDK3* | [22-24] |
| chr18 | q12.1 | Dup | *MEP1B* | [25] |
| chr21 | q21.3 | Del | C21orf7, C21orf109, *BACH1*, C21orf41, ***GRIK1*** | [26-28] |
| chr21 | q22.3 | Dup | C21orf57, *C21orf58*, PCNT, DIP2A | [29] |
| chrX | q13.1 | dup | FAM155B, ***EDA*** | [30,31] |
| chrX | p22.31 | dup | HDHD1, ***STS***, VCX, PNPLA4 | [32-34] |

*Legend: genes already reported to be altered in cancer pathways;* ***genes implicated in breast/ovarian cancer.***

References

1. Bos PD, Zhang XH, Nadal C *et al.* **Genes that mediate breast cancer metastasis to the brain**. *Nature* 2009; 459(7249):1005-9.

2. Oster B, Thorsen K, Lamy P *et al.* **Identification and validation of highly frequent CpG island hypermethylation in colorectal adenomas and carcinomas**. *Int J Cancer* 2011.

3. Gu J, Ajani JA, Hawk ET *et al.* **Genome-wide catalogue of chromosomal aberrations in barrett's esophagus and esophageal adenocarcinoma: a high-density single nucleotide polymorphism array analysis**. *Cancer Prev Res (Phila)* 2010; 3(9):1176-86.

4. Onodera Y, Hashimoto S, Hashimoto A *et al.* **Expression of AMAP1, an ArfGAP, provides novel targets to inhibit breast cancer invasive activities**. *EMBO J* 2005; 24(5):963-73.

5. Hawes SE, Stern JE, Feng Q *et al.* **DNA hypermethylation of tumors from non-small cell lung cancer (NSCLC) patients is associated with gender and histologic type**. *Lung Cancer* 2010; 69(2):172-9.

6. Chattopadhyay I, Singh A, Phukan R *et al.* **Genome-wide analysis of chromosomal alterations in patients with esophageal squamous cell carcinoma exposed to tobacco and betel quid from high-risk area in India**. *Mutat Res* 2010; 696(2):130-8.

7. Nakano H, Miyazawa T, Kinoshita K, Yamada Y, Yoshida T. **Functional screening identifies a microRNA, miR-491 that induces apoptosis by targeting Bcl-X(L) in colorectal cancer cells**. *Int J Cancer* 2010; 127(5):1072-80.

8. Venkatachalam R, Verwiel ET, Kamping EJ *et al.* **Identification of candidate predisposing copy number variants in familial and early-onset colorectal cancer patients**. *Int J Cancer* 2010.

9. Bialek J, Kunanuvat U, Hombach-Klonisch S *et al.* **Relaxin enhances the collagenolytic activity and in vitro invasiveness by upregulating matrix metalloproteinases in human thyroid carcinoma cells**. *Mol Cancer Res* 2011; 9(6):673-87.

10. Brandt B, Roetger A, Bidart JM *et al.* **Early placenta insulin-like growth factor (pro-EPIL) is overexpressed and secreted by c-erbB-2-positive cells with high invasion potential**. *Cancer Res* 2002; 62(4):1020-4.

11. Brandt B, Kemming D, Packeisen J *et al.* **Expression of early placenta insulin-like growth factor in breast cancer cells provides an autocrine loop that predominantly enhances invasiveness and motility**. *Endocr Relat Cancer* 2005; 12(4):823-37.

12. Janneau JL, Maldonado-Estrada J, Tachdjian G *et al.* **Transcriptional expression of genes involved in cell invasion and migration by normal and tumoral trophoblast cells**. *J Clin Endocrinol Metab* 2002; 87(11):5336-9.

13. Kietz S, Feng S, Agoulnik A, Hombach-Klonisch S. **Estrogen and TCDD influence RLN2 gene activity in estrogen receptor-positive human breast cancer cells**. *Ann N Y Acad Sci* 2009; 1160:367-73.

14. Seibold P, Hein R, Schmezer P *et al.* **Polymorphisms in oxidative stress-related genes and postmenopausal breast cancer risk**. *Int J Cancer* 2010.

15. Pylvas M, Puistola U, Kauppila S, Soini Y, Karihtala P. **Oxidative stress-induced antioxidant enzyme expression is an early phenomenon in ovarian carcinogenesis**. *Eur J Cancer* 2010; 46(9):1661-7.

16. Seibold P, Hein R, Schmezer P *et al.* **Polymorphisms in oxidative stress-related genes and postmenopausal breast cancer risk**. *Int J Cancer* 2010.

17. Agundez M, Grau L, Palou J *et al.* **Evaluation of the Methylation Status of Tumour Suppressor Genes for Predicting Bacillus Calmette-Guerin Response in Patients With T1G3 High-Risk Bladder Tumours**. *Eur Urol* 2011; 60(1):131-40.

18. Bol GM, Suijkerbuijk KP, Bart J *et al.* **Methylation profiles of hereditary and sporadic ovarian cancer**. *Histopathology* 2010.

19. Hutajulu SH, Indrasari SR, Indrawati LP *et al.* **Epigenetic markers for early detection of nasopharyngeal carcinoma in a high risk population**. *Mol Cancer* 2011; 10:48.

20. Leong KJ, Wei W, Tannahill LA *et al.* **Methylation profiling of rectal cancer identifies novel markers of early-stage disease**. *Br J Surg* 2011; 98(5):724-34.

21. Benzinger A, Muster N, Koch HB, Yates JR, III, Hermeking H. **Targeted proteomic analysis of 14-3-3 sigma, a p53 effector commonly silenced in cancer**. *Mol Cell Proteomics* 2005; 4(6):785-95.

22. Cho YY, Tang F, Yao K *et al.* **Cyclin-dependent kinase-3-mediated c-Jun phosphorylation at Ser63 and Ser73 enhances cell transformation**. *Cancer Res* 2009; 69(1):272-81.

23. Loi TH, Campain A, Bryant A *et al.* **Discriminating lymphomas and reactive lymphadenopathy in lymph node biopsies by gene expression profiling**. *BMC Med Genomics* 2011; 4:27.

24. McMillin DW, Delmore J, Negri J *et al.* **Molecular and cellular effects of multi-targeted cyclin-dependent kinase inhibition in myeloma: biological and clinical implications**. *Br J Haematol* 2011; 152(4):420-32.

25. Jiang W, Kumar JM, Matters GL, Bond JS. **Structure of the mouse metalloprotease meprin beta gene (Mep1b): alternative splicing in cancer cells**. *Gene* 2000; 248(1-2):77-87.

26. Miyazaki T, Kirino Y, Takeno M *et al.* **Expression of heme oxygenase-1 in human leukemic cells and its regulation by transcriptional repressor Bach1**. *Cancer Sci* 2010; 101(6):1409-16.

27. Murabito JM, Rosenberg CL, Finger D *et al.* **A genome-wide association study of breast and prostate cancer in the NHLBI's Framingham Heart Study**. *BMC Med Genet* 2007; 8 Suppl 1:S6.

28. Warnatz HJ, Schmidt D, Manke T *et al.* **The BTB and CNC Homology 1 (BACH1) Target Genes Are Involved in the Oxidative Stress Response and in Control of the Cell Cycle**. *J Biol Chem* 2011; 286(26):23521-32.

29. Hanafusa T, Mohamed AE, Kitaoka K *et al.* **Isolation and characterization of human lung cancer antigens by serological screening with autologous antibodies**. *Cancer Lett* 2011; 301(1):57-62.

30. Punj V, Matta H, Chaudhary PM. **X-linked ectodermal dysplasia receptor is downregulated in breast cancer via promoter methylation**. *Clin Cancer Res* 2010; 16(4):1140-8.

31. Tanikawa C, Ri C, Kumar V, Nakamura Y, Matsuda K. **Crosstalk of EDA-A2/XEDAR in the p53 signaling pathway**. *Mol Cancer Res* 2010; 8(6):855-63.

32. Fu J, Weise AM, Falany JL *et al.* **Expression of estrogenicity genes in a lineage cell culture model of human breast cancer progression**. *Breast Cancer Res Treat* 2010; 120(1):35-45.

33. Serrano A, Lethe B, Delroisse JM *et al.* **Quantitative evaluation of the expression of MAGE genes in tumors by limiting dilution of cDNA libraries**. *Int J Cancer* 1999; 83(5):664-9.

34. Subramanian K, Jia D, Kapoor-Vazirani P *et al.* **Regulation of estrogen receptor alpha by the SET7 lysine methyltransferase**. *Mol Cell* 2008; 30(3):336-47.
